# Brain Structures, Circuits, and Networks Involved in Immune Regulation, Periodontal Health, and Disease

**DOI:** 10.3390/life15101572

**Published:** 2025-10-09

**Authors:** Torbjørn Jarle Breivik, Per Gjermo, Per Kristian Opstad, Robert Murison, Stephan von Hörsten, Inge Fristad

**Affiliations:** 1 Institute of Clinical Odontology–Periodontology, Faculty of Dentistry, University of Oslo, 0455 Oslo, Norway; breivik.torbjorn@gmail.com (T.J.B.);; 2 Norwegian Defense Research Establishment, Division for Protection, 2027 Kjeller, Norway; 3 Department of Biological and Medical Psychology, Faculty of Psychology, University Bergen, 5021 Bergen, Norway; 4 Department of Experimental Therapy, Preclinical Experimental Center, University Hospital Erlangen, Friedrich-Alexander-Universität Erlangen-Nürnberg (FAU), 91054 Erlangen, Germany; 5 Department of Clinical Dentistry, Faculty of Medicine, University of Bergen, 5009 Bergen, Norway

**Keywords:** microbiota–immune–brain-interaction, brain areas, circuits, networks, neuroinflammation, stress coping, gingival health, gingivitis, periodontitis

## Abstract

The interaction between microorganisms in the dental microfilm (plaque) at the gingival margin, the immune system, and the brain is vital for gingival health. The brain constantly receives information regarding microbial composition and inflammation status through afferent nerves and the bloodstream. It modulates immune responses via efferent nerves and hormonal systems to maintain homeostasis. This relationship determines whether the gingiva remains healthy or develops into gingivitis (non-destructive inflammation) or periodontitis (a destructive condition), collectively referred to as periodontal disease. Factors associated with severe periodontitis heighten the responsiveness of this homeostatic system, diminishing the adaptive immune system’s defence against symbiotic microorganisms with pathogenic properties, known as pathobionts. This leads to excessive innate immune system activation, effectively preventing infection but damaging the periodontium. Consequently, investigating the microbiota–brain axis is vital for understanding its impact on periodontal health and disease. Herein, we examine recent advancements in how the defence against pathobionts is organised within the brain, and how it regulates and adapts the pro-inflammatory and anti-inflammatory immune balance, controlling microbiota composition. It also discussed how pathobionts and emotional stress can trigger neurodegenerative diseases, and how inadequate coping strategies for managing daily stress and shift work can disrupt brain circuits linked to immune regulation, weakening the adaptive immune response against pathobionts.

## 1. Introduction

The last 30 years of interdisciplinary research on bidirectional immune–brain communication and microbiota–immune–brain interaction have increased our understanding of how genetics and environmental factors contribute to the development and progression of inflammatory diseases [1,2,3,4,5], including those triggered by the microbiota [6,7,8,9,10]. This research has revealed an intricate connection between the microbiota, the immune system, and the brain, governed by the autonomic equilibrium-regulating (homeostatic) nervous and hormonal systems, collectively referred to as the stress system. The stress system is an innate, stereotypical, adaptive response to danger signals (stressors) that has evolved to restore the non-stressed homeostatic set point. Although essential for survival, it can be misguided or dysregulated by internal and external environments, resulting in disease [11,12]. This system regulates and coordinates the body’s overall equilibrium (homeostasis), including heart rate, blood pressure, core temperature, blood sugar levels, appetite, emotional behaviour, and immune responses [11,12,13,14]. In immune regulation, one of its primary functions is to dampen strong pro-inflammatory cytokine responses, including tumour necrosis factor (TNF)-α. This immune mediator promotes adaptive T helper 1 (Th1) and Th17 responses, which defend against intracellular and extracellular pathogenic microorganisms, respectively [14]. In this way, the stress system skews the T helper 1 (Th1)/Th2 balance and the Th17/T-regulatory (Treg) balance towards the anti-inflammatory Th2 and Treg direction, protecting organisms against “overshooting” pro-inflammatory responses. These balances are crucial for managing the diverse microorganisms in the microbiota [15,16,17], and consequently for overall health and disease [18].

In experimental models, it has been demonstrated that animals with sustained, overly strong stress responses are more prone to experimental periodontitis, whereas stress hypo-responders are more resistant. This phenomenon was observed regardless of whether the high- or low-stress response was genetically determined [19,20,21], permanently altered by very early life experiences through epigenetic mechanisms [22,23,24,25,26], or influenced in adulthood by factors and diseases associated with severe human periodontitis [27,28,29,30,31]. It is also demonstrated that immune stimuli, which drive the Th1/Th2 balance and the Th17/Treg balance in a pro-inflammatory (Th1 and Th17) direction, control the growth of pathobionts, thereby inhibiting the progression of the disease [18,32,33,34]. In addition, factors and diseases associated with severe periodontitis in humans skewed this balance in the opposite direction by enhancing the responsiveness of the stress system [27,28,29,30,31]. These findings are crucial for understanding gingivitis and periodontitis, collectively referred to as periodontal diseases [18]. Also, pharmacological substances may impair homeostasis. For example, drugs that inhibit or block signalling in sensory neurons, or the binding of stress mediators to their receptors, such as beta-blockers and RU 486 (mifepristone) [18,27], inhibit experimental periodontitis in rats, whereas drugs that bind to and stimulate these receptors enhance experimental periodontal tissue destruction, as discussed in Section 2 and Section 3.6. Interestingly, nicotine was found to be an exception: it binds to specific acetylcholine receptors (AchRs), known as nicotine AchRs (nAchRs), present on cells in the central nervous system and on immune cells, reducing the production and release of TNF-α. Consequently, nicotine exacerbates susceptibility to periodontitis by inhibiting TNF-α responses, thus diminishing the immunological defence against pathobionts [18,29].

In a recent review article, we addressed the complex relationships between dental plaque microorganisms, the immune system, and the brain as a framework for understanding periodontal health and disease [18]. Why do some people experience severe periodontitis, whereas the majority do not develop advanced periodontal disease, even in the absence of optimal oral hygiene? This narrative review further addresses these connections by integrating previous and present findings within periodontology, gastroenterology, immunology, neuroendocrinology, and brain research. It highlights recent discoveries on how the immune system manages pathobionts in dental biofilms at the gingival margin, how the brain is informed about the microbiota’s composition and the inflammatory response, how these processes are coordinated and regulated within specific brain regions, circuits, and networks, and finally, how the brain modulates these responses through equilibrium-maintaining nervous, neuroendocrine, and purely endocrine (hormonal) systems. This review also considers how infections caused by pathobionts and external pathogens, along with chronic emotional stress, can trigger a depressive mood and contribute to neurodegenerative diseases such as Alzheimer’s. The depressive mood caused by pathogens is part of a coordinated set of adaptive behavioural changes, known as sickness behaviour, that occurs during infection. It further emphasises how inadequate coping strategies for managing daily stress, shift work, loss of sleep, and sleep deprivation (poor sleep quality) may disrupt the bidirectional microbiota–brain communication, resulting in a weakened immune response and susceptibility to periodontitis. As the interplay between these systems is crucial for understanding both resistance to and susceptibility to periodontal disease, we finally address implications of this bidirectional communication between dental microflora and the brain in the prevention and treatment of periodontitis.

## 2. The Dental Microflora-Brain Axis

**Figure 1:**
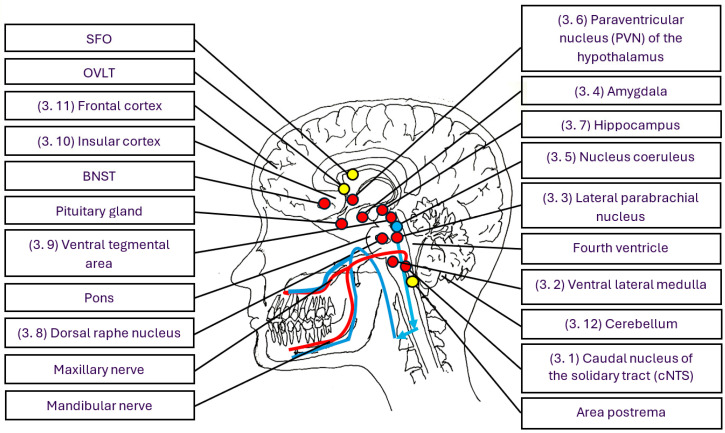
The figure illustrates brain areas that regulate and coordinate immune responses, managing microbial load and bacterial composition in the dental microfilm (plaque) at the gingival margin. The numbers (3.1–3.12) refer to the text sections discussing their function. The red lines indicate sensory nerves, transmitting information to the caudal nucleus tractus solitarius (cNTS). The sensory circumventricular organs (COVs), which receive information from the blood during infections, are marked as yellow spots and positioned around the third and fourth ventricles. Together with the sensory peptidergic nerves, the COV, known as the area postrema, integrates information about the inflammatory state, microbial load, and bacterial composition from periodontal tissues and associated immune organs into the cNTS. This, in turn, activates other brain structures, circuits, and networks (red spots) that ultimately stimulate the efferent (brain-to-periphery signalling) nervous and hormonal systems, referred to as the stress system. The stress system is an equilibrium-regulating (homeostatic) nervous and hormonal system that returns information to periodontal tissues and associated immune organs, thereby regulating the activity of immune cells. The blue lines illustrate sympathetic nerves originating from noradrenaline-producing neurons in the superior cervical ganglion, with synaptic connections to the locus coeruleus (indicated by the blue spot). From the sympathetic ganglion these nerves run along the spinal cord, before they follow the maxillary and mandibular nerves and blood vessels to oral tissues and corresponding immune organs. The sympathetic nervous system is considered one of the most essential brain-controlled immunoregulatory mechanisms within the stress system. The organum vasculosum of the lamina terminalis (OVLT), and the subfornical organ (SFO) are the COVs that trigger fever and initiate sickness behaviour following infections, respectively. The bed nucleus of the stria terminalis (BNST) is a brain structure vital for activating stress responses and despair-like behaviour, including feelings of hopelessness, helplessness, and profound sadness under severe emotional stress and intense infections.

**Figure 2:**
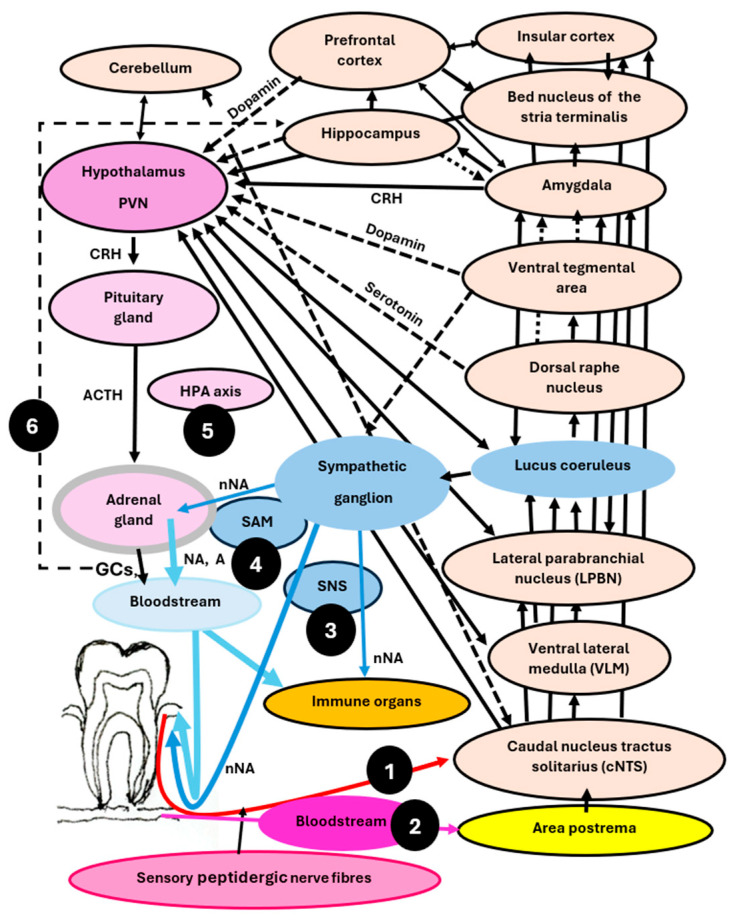
The illustration depicts the organisation of brain areas, circuits, and networks involved in overarching immune regulation, referred to as the dental plaque-brain axis. (1) Sensory information regarding microorganisms in the dental plaque and the inflammation they induce in the gingiva, reaches the brain’s caudal nucleus tractus solitarius (cNTS) via sensory nerve fibres (red arrow) in the maxillary and mandibular nerves, and (2) through the bloodstream via the area postrema during infections, as illustrated with pink arrow. The brain processes this information by interacting with other brain structures and activates the stress system, which comprises (3) the sympathetic nervous system (SNS), (4) the sympathetic-adrenal-medullary system (SAM), and (5) the hypothalamic–pituitary–adrenal (HPA) axis. Sympathetic nerves (blue arrows) originate from the cervical sympathetic ganglion. The locus coeruleus (the blue spot) sends signals to the sympathetic ganglion, which then transmits signals to the gingiva and immune organs, releasing neurotransmitters, mainly noradrenaline (nNA), from their nerve endings. Sympathetic nerves innervating the medulla of the adrenal glands (the SAM) trigger the release of the hormones noradrenaline (NA) and adrenaline (A) into the bloodstream. The HPA axis (5) is a purely hormonal system in which neurons in the paraventricular nucleus (PVN) of the hypothalamus produce the neuropeptide hormone corticotropin-releasing hormone (CRH), which stimulates cells in the pituitary gland to produce adrenocorticotropic hormone (ACTH). This hormone then reaches the adrenal cortex via the bloodstream, where it stimulates cells to produce glucocorticoid hormones (GCs) that are released into the bloodstream. Pathway (6) illustrates a negative feedback loop (dotted arrow) where glucocorticoids inhibit HPA axis activation, thus reducing further GC release.

The dental microflora-brain axis refers to the bidirectional communication network between the microorganisms in dental plaque at the gingival margin and the brain (Figure 1 and Figure 2). This network helps coordinate and regulate gingival immune responses, as well as the quantity and composition of dental plaque [18]. The bidirectional communication network between the gut microbiota and the brain is known as the microbiota–gut–brain axis or the brain–gut–microbiota axis [6,7,8,9,10,35,36,37,38,39,40,41]. The communication network between the body and the brain, known as the body–brain axis [42], is a main conductor of an organism’s physiology and is essential for survival. It allows the brain to process and respond to internal and external signals, distinguishing between harmful and benign stimuli, thereby enabling the organism to adapt accordingly [11,12,18,43,44,45,46].

The information from the microorganisms in microbiota, and the inflammatory state they induce in mucosal tissues, is constantly relayed to the brain via specialised sensory nerve fibres [4,5,6,7,8,9,10,18]. These nerves are called sensory peptidergic nerves because they release neuropeptides with pro-inflammatory and vasoactive functions, including substance P (SP) and calcitonin gene-related peptide (CGRP) at their nerve terminals (endings) [47,48,49]. They are also known as nociceptive and capsaicin-sensitive nerves, due to their pain response to harmful stimuli and their extreme sensitivity to capsaicin (the spicy substance in chilli peppers) [47,48,49,50,51,52]. Their nerve endings have receptors sensing components from microorganisms, such as the Toll-like receptors (TLR)-4 that detect cell wall component lipopolysaccharides (LPS) of Gram-negative bacteria, including *P. gingivalis* [50], and sensors for immune mediators like TNF-α, interleukin (IL)-1β, IL-6, IL-17, and prostaglandin E2 (PGE2) receptors. Blocking or inhibiting the neurotransmission (signalling) in these nerves reduces stress responses and inhibits the development of periodontitis [53,54,55]. In contrast, chronic pain increases signalling in these nerves, worsening periodontal breakdown and influencing various mediators involved in tissue damage and repair, thus worsening periodontal breakdown [56].

Sensory peptidergic nerve fibres in the oral cavity are part of the maxillary and mandibular nerves [18]. Together with the ophthalmic nerve, they form the trigeminal nerve, which enters the brain on each side of the midpoint of the brainstem, known as the pons, as illustrated by the red lines in Figure 1. During local and systemic infections, information about microbial products and the immune mediators they trigger also reaches the brain via the blood and sensory circumventricular organs (CVOs), highlighted as yellow spots in Figure 1. COVs are specialised brain regions around the third and fourth ventricles, i.e., cavities filled with cerebrospinal fluid that lack a strict blood–brain barrier. Microbial products and immune mediators from the blood are continuously monitored in these organs during health and disease [56,57]. They are categorised into a sensory group, including the area postrema, the subfornical organ (SFO), and the organum vasculosum of the lamina terminalis (OVLT) (Figure 1), which conveys information from the blood to the brain, and a secretory group. The secretory COVs send peptide hormones and other mediators from the brain to the bloodstream, regulating the activity of various body organs. Although sensory CVOs lack an endothelial barrier, this does not imply unrestricted movement of blood-derived molecules into the CVO parenchyma. Instead, a dense protective barrier is formed by non-neural cells, astrocytes and tanycytes, which express sensors that detect microbial products and immune mediators in the blood. The area postrema, a paired structure in the medulla oblongata (Figure 1 and Figure 2), monitors microorganisms, microbial products, and immune mediators that drive the stress system during systemic infections. Like the information from the sensory peptidergic nerves, the data from the area postrema activate neurons in the brainstem that consistently provide the autonomic-affective functional basis for the homeostatic control of the entire body [57,58].

This phenomenon is feasible because the sensory peptidergic nerves and the area postrema have sensors capable of detecting and distinguishing between various types of microorganisms and immune mediators present in the blood and dental plaque at the gingival margin. This enables the brain to recognise the inflammatory condition in the gingiva and blood, the composition of microorganisms in the microbiota, including those in the dental plaque at the gingival margin, and the status of body temperature and various other bodily functions [56,57,58]. For example, the microbial products and immune mediators, reaching the brain via the area postrema during an infection, stimulate neurons in the paraventricular nucleus of the hypothalamus, driving the stress system, while those reaching the brain via the OVLT stimulate hypothalamic neurons in the preoptic area, which raises core temperature and triggers fever [58,59,60]. Fever supports the immune response by recruiting immune cells and boosting their effectiveness against pathogens and cancer cells. This is a part of the adaptive response to an infection that aids the organism in surviving, which, as mentioned above, is known as sickness behaviour [2]. Common symptoms of systemic infection/sickness behaviour have much in common with typical (melancholic) depression, including lethargy, decreased appetite, weight loss, and increased pain sensitivity [2]. The SFO (Figure 1 and Figure 2) mediates these symptoms. Additionally, this sensory organ responds to immune signals from the bloodstream via TLRs, cytokines and prostaglandin receptors. The SFO influences emotional responses via projections to the bed nucleus of the stria terminalis (BNST; Figure 1) and is vital for activating the stress response under emotional stress [59] and LPS-induced despair-like behaviour [61].

After being integrated into functional outputs within the brain, information is relayed back to peripheral immunoinflammatory processes through the nervous and hormonal systems, collectively known as the stress system, which comprises the sympathetic nervous system (SNS), the sympathetic-medullary system (SAM), and the hypothalamic–pituitary–adrenal (HPA) axis (Figure 2) [1,2,3,4,5]. These systems maintain homeostasis by orchestrating the activity in various organs. The sympathetic nerves innervating the gingiva arise from noradrenergic neurons in the sympathetic cervical ganglion (SCG) that travel along the maxillary and mandibular nerve branches and blood vessels (Figure 1) [62]. The sympathetic nerves that activate the SAM and release the immunoregulatory hormones noradrenaline (NA) and adrenaline (A) from the adrenal medulla originate from the splenic nerve [63].

Effective regulation of immunoinflammatory responses by the brain’s stress system is essential for health, as improper regulation may lead to disease [11,12,45,46]. During active periodontitis, symbiotic microorganisms (symbionts) with invasive properties, referred to as pathobionts to distinguish them from external pathogens, dominate the subgingival dental plaque. This includes Gram-negative bacteria such as *Porphyromonas (P.) gingivalis*, *Tannerella forsythensis*, *Aggregatibacter (A.) actinomycetemcomitans*, and the Gram-negative spirochete *Treponema denticola*, along with certain common fungi and viruses. In contrast, non-invasive symbionts, including Gram-positive bacteria, dominate in gingivitis [64,65,66,67]. Microbial imbalance in gingivitis or periodontitis is called dysbiosis [68,69,70]. Therefore, understanding the mechanisms that influence the growth of pathobionts, as well as how risk factors for severe periodontitis may promote their growth and contribute to dysbiosis, is essential for the development and progression of periodontitis. Thus, the balance of microorganisms in the microbiota is regulated by the interaction between pro-inflammatory and anti-inflammatory immune responses, encompassing the Th1/Th2 and Th17/Treg balances [17,18,71,72,73], which are further governed by the brain’s stress system [11,12,18].

## 3. Brain Structures, Circuits, Networks, and Their Function in Immune Regulation, Periodontal Health, and Disease

A standard approach when studying how sensory nerve endings and COVs detect pathobionts and immune products, as well as how they activate brain structures and the circuits and networks they trigger, involves exposing tissues to Gram-negative bacterial LPS, either through local or systemic injections. This simulates both local and systemic bacterial infections and enables monitoring of brain structure activities. Since systemic LPS triggers a range of sickness-induced behavioural changes, including symptoms similar to depression induced by emotional stress, it also aids in investigating mechanisms and treatments for this condition [74,75,76]. Functional magnetic resonance imaging (fMRI) has revealed how infection-induced brain activity affects the nervous and hormonal systems involved in health and disease [77]. Immunoinflammatory responses and emotional stress, in addition to activating brain neurons, activate non-neuronal cells named glial cells [75,76]. These cells communicate by releasing cytokines, and activation of these cells is, as discussed in Section 4, a part of normal regulatory processes in the brain. However, if chronic and excessive, it can trigger destructive inflammatory responses and initiate depressive moods and other symptoms of sickness behaviour [2], and neurodegenerative conditions like Alzheimer’s disease [76,78].

The sensory peptidergic nerves and sensory CVOs (area postrema, OVLT, and SFO), which bring information from the dental plaque at the gingival margin and the inflammatory state in the gingiva and blood to the brain, are connected to brain areas that regulate physical and emotional stress, behaviour, pain, immune responses and several other physiological responses [1,2,3,4,5,61]. These regulatory systems in the brain are linked to the development of periodontitis [18]. Other factors linked to severe periodontitis, such as shift work, loss of sleep, sleep deprivation, and poor coping behaviours, also contribute to the development of periodontitis, as discussed in Section 3.11 and Section 4. Key brain structures involved in stress, immune regulation, and associated diseases are illustrated in Figure 1 and Figure 2.

### 3.1. The Caudal Nucleus of the Nucleus Tractus Solitarius (cNTS)—An Essential Node Processing Information from the Gingiva and Activating the Stress System

A major first stop for incoming information from oral tissues to the brain, carried by sensory fibres in the trigeminal nerve and via the area postrema, is called the caudal nucleus tractus solitarius (cNTS) (Figure 2) [79,80,81]. By direct and indirect activation of the efferent immunoregulatory stress system, the cNTS appears to be the primary region in the brain that processes incoming information regarding microorganisms in microbiota and the inflammatory status in mucosal tissues [81,82,83,84]. Recent animal studies have shown that sensory peptidergic nerves and sensory CVOs carry pro-inflammatory and anti-inflammatory signals to the cNTS [83]. Both signals reduce the release of pro-inflammatory cytokines and promote anti-inflammatory cytokines when Gram-negative bacteria or other pathogens are detected. Pro-inflammatory cytokines adversely influence pro-inflammatory immune responses through negative feedback, whereas the anti-inflammatory cytokine IL-10 exerts a similar effect via positive feedback to the brain. Chemical suppression of cNTS neurons in animal models was shown to increase TNF-α levels threefold while IL-10 levels decreased tenfold. Conversely, artificial activation of cNTS neurons reduced pro-inflammatory cytokines by 70% and increased IL-10 tenfold [83], thus demonstrating that the central nervous system can significantly influence immune responses in peripheral tissues, not just fine-tuning them. The experiment also demonstrated that strong incoming danger signals from the periphery to the brain shift the pro-inflammatory/anti-inflammatory balance towards the anti-inflammatory side.

Gram-negative bacterial LPS activates brain neurons in the cNTS, known as ADCYAP1+ neurons, which are responsible for various aspects of sickness behaviour [85]. Activation of ADCYAP1+ neurons in cNTS helps the body to manage infections by inducing common illness symptoms such as chills, fever, pain, fatigue, loss of appetite, and social withdrawal. The strength of the stress response, immune responses, and sickness symptoms partly depends on the information transmitted to the brain via cNTS [2,82]. Chemical blockade of sensory peptidergic nerves in rats has been found to reduce the stress hormone corticosterone serum levels in response to systemic LPS stimulation, followed by a pro-inflammatory/anti-inflammatory balance shift, significantly inhibiting the progression of experimental periodontitis [53]. The pro-inflammatory cytokine TNF-α (and IL-12) plays a key role in mediating inflammatory responses that promote protective adaptive responses (Th1 and Th17) against pathobionts [14], thus inhibiting the progression of periodontitis [18]. Individuals with insufficient stress responses to Gram-negative bacteria or LPS develop excessive TNF-α responses. Although robust TNF-α responses are vital for combating Gram-negative bacteria, an excessive TNF-α response can lead to harmful consequences, including autoimmune diseases and potentially fatal conditions, such as sepsis, septic shock, tissue damage, and death [38,39,40,41,86].

Gram-negative bacteria are sensed in the brain based on their toxicity, mainly influenced by the lipid A structure in LPS molecules and their ability to bind to TLR4, affecting the pro-inflammatory cytokine release, mainly TNF-α [81]. For instance, LPS from Gram-negative gut bacteria *Escherichia* (*E.*) coli, the most dangerous bacterial molecule described, triggers strong TNF-α responses and activates neurons in the sensory area postrema, OVALT, and SFO, as well as the secretory median eminence, making it more dangerous than LPS from the periodontal Gram-negative pathobiont *P. gingivalis* [81]. Consequently, abnormal growth of *E. coli* in the gut is likely more detrimental and responsible for tissue-destructive inflammatory reactions in the brain than the abnormal growth of *P. gingivalis* in dental plaque.

Together, these studies demonstrate that the cNTS is the initial step in acquiring information about microorganisms and inflammation from the gingiva to the brain. This information is processed in this nucleus, and the activation strength of cNTS neurons significantly influences both direct and indirect efferent stress responses. The extent to which an individual reacts to environmental stress, be it suitably, insufficiently, or excessively, is vital for the outcomes of an immune response, the composition of the microbiota, and periodontal health [18].

### 3.2. The Ventral Lateral Medulla—Regulating and Coordinating Stress and Immune Responses

Systemic LPS activates neurons in the ventral lateral medulla (VLM) of the brainstem (Figure 1 and Figure 2), which consists of the caudal (cVLM) and rostral (rVLM) parts [87,88]. The cVLM processes inflammatory signals from the cNTS, relaying this information to the parvocellular neurons in the paraventricular nucleus (PVN) of the hypothalamus, driving the HPA axis (Figure 2). The PVNs have projections to the rVLM, which activates noradrenaline-producing neurons in the brain stem, regulating the activity of the sympathetic nervous systems (SNS and SAM) [89]. Thus, during stressful stimuli the hypothalamic PVN neurons drive the HPA axis and the rest of the stress response system.

### 3.3. The Lateral Parabrachial Nucleus—Serving as a Hub, Linking Information to Other Brain Regions Involved in Stress and Immune Regulation

The lateral parabrachial nucleus (LPBN) is located in the dorsal vagal complex of the pons and is part of a group of nuclei called the parabrachial nucleus complex (Figure 1 and Figure 2) [90,91,92]. It consists of three main neuronal groups. Systemic immune stimuli activate these neurons, but mainly in the lateral division [92]. These neurons collect data on inflammation and infections from sensory nerves and the blood via the cNTS, and are connected to brain regions that regulate stress and immune responses [93]. By the signalling molecule CGRP (calcitonin gene-related peptide), the parabrachial nucleus may function as a general alarm and appears to be an essential link or hub connecting information about microorganisms and the inflammatory state to the brain, to induce a stress response [91]. The lateral parabrachial nucleus is vital for LPS-induced fever, enhancing immune cell effectiveness during infections [2,92]. Systemic LPS-induced activation of neurons in the cNTS, extending to the parabrachial nucleus, also promotes increased sleep behaviour, facilitating recovery from illness [93].

### 3.4. The Amygdala—Alarming the Body When Sensing Pathobionts and Any Other Danger Signals

The amygdala is a paired, almond-shaped structure located in the middle of the brain. (Figure 1). It plays a crucial role in regulating fear, stress, immune responses, and emotions through its connections by linking the amygdala to the hypothalamus [94,95,96,97]. It receives information about microbial composition and inflammation from the LPBN and cNTS, which gathers data from the gingiva through sensory peptidergic nerves and the bloodstream via the CVO area postrema. Thus, the amygdala acts as the brain’s major alarm centre for fear and related emotions induced by acute and chronic stress, depression [93], and danger signals from pathogens [94]. Additionally, the amygdala connects with several brain regions to orchestrate an appropriate stress response [95].

The amygdala comprises over 13 subnuclei, each activated by distinct danger signals, and regulates emotional memory, mood disorders, anxiety, and cognitive functions [95]. Exposure to Gram-negative bacteria or LPS from these bacteria activates neurons in the central amygdala, mainly through the pro-inflammatory cytokine IL-1β [95,96,97]. Neurons in the anterior basolateral amygdala respond to negative emotions, whereas those in the posterior region respond to positive emotions [98]. Consequently, it is probable that the amygdala processes information regarding invasive Gram-negative bacteria and other pathobionts in one area while handling Gram-positive bacteria and other non-invasive symbionts in another.

The medial amygdala links the amygdala to the hypothalamus, facilitating social rewards by interacting with serotonin-producing neurons in the dorsal raphe nucleus and dopamine-producing neurons in the ventral tegmental area (Figure 2) [97,98,99,100]. As explained in Section 3.8, serotonin-producing neurons in the dorsal raphe nucleus and dopamine-producing neurons in the ventral tegmental area are inhibited in individuals with poorly developed coping behaviour, a significant risk factor in periodontitis. In addition, high serotonin levels induce positive feelings, and increased serotonin levels reduce stress responses. The weaker stress response shifts the immune Th1/Th2 balance and the Th17/Treg balance towards the pathobionts-protective pro-inflammatory Th1 and Th17 sides, reducing the susceptibility to experimental periodontitis in rats. In contrast, lower serotonin levels, typically seen in people exposed to severe emotional stress and depression of the melancholic type, shift the balance towards the Th2 and Treg side. Stimulating dopamine neurons in the ventral tegmental area also enhances positive emotions. In addition, it strengthens immunity against Gram-negative bacteria by inhibiting noradrenaline release from sympathetic nerve endings in the bone marrow.

Information regarding Gram-negative bacteria and other invasive microorganisms activates neurons in the amygdala, releasing CRH (the corticotropin-releasing hormone). This peptide hormone stimulates neurons in various brain regions, including the hypothalamus (Figure 2). The hypothalamic CRH activates the HPA axis to release glucocorticoid (GC) from the adrenal cortex and stimulates the locus coeruleus to trigger the sympathetic system, releasing the neurotransmitter noradrenaline from sympathetic nerve endings and the hormones noradrenaline and adrenaline from the adrenal medulla. Furthermore, the amygdala transmits signals to the hippocampus (Figure 2). The number of glucocorticoid receptors (GRs) in the brain, particularly in the hippocampus, plays a significant role in the strength of the stress response and susceptibility to gingivitis and periodontitis [18]. Early life experiences can down- and up-regulate the expression of these receptors via epigenetic mechanisms, making an individual both more susceptible and more resistant to periodontitis, respectively [18].

### 3.5. The Locus Coeruleus—Driving the Sympathetic Component of the Stress System, Crucial for Brain-Controlled Efferent Immune Regulation

The locus coeruleus (Figure 1 and Figure 2) is a small but prominent nucleus, producing noradrenaline. It is a stress-sensitive brain region crucial in stress response and immune regulation [101]. Signals of danger, whether from emotional, physical, or immunological/infectious sources, activate these noradrenaline-producing neurons. The stress LC-NE (locus coeruleus-noradrenaline) system receives afferent projections from a wide range of regions and sends projections to almost all brain areas involved in stress and immune regulation (Figure 2) [101,102,103,104,105,106]. This induces anxiety [102], and by activating the peripheral arm of the stress locus coeruleus-noradrenaline system, noradrenaline (nNA) from sympathetic endings and the hormones noradrenaline (NA) and adrenaline (A) from the adrenal medullae regulate most body functions, including local and systemic immune responses [103,104,105,106,107,108,109,110,111,112,113]. NA and A (along with dopamine) are classified as catecholamines. Like nNA, NA and A bind to sensors in the cell membranes of immune cells, known as adrenergic receptors. These receptors are categorised into two groups, α and β, and are further divided into nine subtypes (α1A, α1B, α1D, and α2A, α2B, α2C, and β1, β2, β3). When binding to α1 or α2 adrenergic receptors on dendritic cells and macrophages, it triggers pro-inflammatory responses at low concentrations and during the early phase of an immunoinflammatory response, while suppressing pro-inflammatory responses via β2-adrenergic receptors at high concentrations and during the late phase. T and B cells primarily express β2-adrenergic receptors. On average, Th1 cells have around 200–400 binding sites per cell, whereas B cells have an even higher expression. When naïve Th0 cells receive signals from antigen-presenting cells about pathogens, and develop into Th1 cells, their β2-receptor expression increases. In contrast, when antigen-presenting cells capture allergens or components from damaged tissues, they migrate to the draining lymph nodes where they present antigenic components to naive Th (Th0) cells that differentiate into Th2 cells. This results in a reduction in β2-receptor expression, which may potentially lead to disrepair. This shift is attributed to epigenetic changes in the β2-adrenergic receptor gene [107]. When an individual is exposed to emotional stressors or pathogens, released noradrenaline and adrenaline enhance anti-inflammatory Th2 responses while suppressing Th1 responses, skewing the Th1/Th2 balance toward the Th2 side. Th17 cells also express β2-adrenergic receptors, and drugs that stimulate these sensors inhibit Th17 responses and skew the Th17/Treg balance in the Treg direction [108,109]. Since Th17 responses coordinate immune responses that prevent pathogenic bacteria and fungi from infecting the gingiva, weak Th17 responses are unfavourable in periodontitis [18]. However, weak Th17 responses are advantageous in pro-inflammatory autoimmune diseases, such as arthritis [108,109]. In an experimental animal model of encephalomyelitis (a model of multiple sclerosis), chronic activation of noradrenergic neurons in the locus coeruleus through chemogenetics reduces the severity of the disease [110]. Thus, what is favourable in pro-inflammatory autoimmune diseases is a disadvantage in periodontitis and vice versa.

The phenomenon, where drugs enhance or inhibit neurotransmission (signalling) in sympathetic nerves and influence disease susceptibility, is also evident in periodontitis [18]. For example, drugs that inhibit or block the ability of noradrenaline and adrenaline to bind to the β2-adrenergic receptors, called beta-blockers, which are commonly used to reduce blood pressure, inhibit experimental periodontitis in rats [18,111]. Likewise, chemical blockade with reduced signalling in sympathetic nerves enhances TNF-α responses to LPS stimulation and inhibits experimental periodontitis [112]. TNF-α drives the Th1/Th2 and Th17/Treg balances in the pro-inflammatory and pathobiont-protective Th1 and Th17 direction [14,85]. α2-adrenergic receptor agonists like Guanabenz also inhibit periodontitis progression [114]. These drugs reduce stress responses and shift immune balances towards pathobiont-protective Th1 and Th17 responses [115].

Like numerous physiological processes in mammals, the secretion of noradrenaline, adrenaline and GC is regulated by a circadian clock in the suprachiasmatic nucleus of the hypothalamus. Through this mechanism, various immune functions follow a circadian rhythmic pattern. Disturbance of these rhythms, such as shift work, enhances stress responses, which in turn affect immune responses, disease susceptibility, and overall health [116].

### 3.6. The Hypothalamus—A Command Centre Regulating Stress and Immune Responses, Playing a Pivotal Role in Periodontal Health and Disease

The hypothalamus, situated above the pituitary gland (Figure 1), is regarded as a primary command centre regulating body homeostasis [117]. It contains a cluster of nuclei that has a variety of functions, including control of body temperature (the supraoptic nucleus) [69,70], appetite (the arcuate nucleus) [118], stress and immune responses (the paraventricular nucleus; PVN) [119,120]. Emotional stress, including severe anxiety and depression [121] and Gram-negative bacterial LPS and cytokines signalled via the cNTS, activate the parvocellular neurons of the PVN in the hypothalamus, resulting in the release of CRH from these neurons [60,61,62]. As illustrated in Figure 2, this peptide hormone stimulates the pituitary gland to secrete the adrenocorticotropic hormone (ACTH), triggering GCs production from the adrenal cortex, which are released into the general blood circulation [11,12].

This hormonal cascade (CRH→ACTH→GC) is known as the HPA axis (Figure 2). Any danger signals, real or imagined, activate the HPA axis [11,12,19]. By activating the CRH-producing parvocellular neurons in the PVN of the hypothalamus, the HPA axis regulates and coordinates stress and immune responses by secreting GC from the adrenal cortexes, which are predominantly cortisol in humans and corticosterone in rodents [1,2,12,18]. Cortisol and corticosterone enter the bloodstream, and in peripheral tissues, these hormones bind to two types of intracellular nuclear receptors, the GRs and the ten times more sensitive mineralocorticoid receptors (MRs), which are expressed in all types of body cells with a nucleus. These receptors regulate genes coding for pro-inflammatory and anti-inflammatory cytokines [5,6,18]. Thus, cortisol and corticosterone are the final effector stress mediators released by the adrenal cortex during the activation of parvocellular neurons in the PVN of the hypothalamus, triggered by exposure to pathobionts in the subgingival dental plaque microfilm, emotional stress, and any other danger signals. In this way, the HPA axis functions as a control system for immune responses, protecting the organism from systemic ‘overshooting’ with pro-inflammatory cytokines during severe inflammation and infection [11,12].

Drugs that bind and activate GRs, such as the synthetic GC dexamethasone, enhance experimental periodontal tissue destruction [122,123]. By binding to GRs, dexamethasone reduces the pro-inflammatory cytokines TNF-α and IL-12 production. These cytokines shift the immune response balance towards pro-inflammatory pathobiont/pathogen-protective adaptive responses (Th1 and Th17) [85]. Thus, dexamethasone treatment enhances the development of periodontitis by dampening pathogen-protective pro-inflammatory Th1 and Th17 responses. In contrast, when a high dose of dexamethasone is given to newborn rats, it permanently reduces their HPA axis responsiveness and makes them more resistant to periodontitis than adults [25]. The same effects are achieved by chronic treatment with a GR antagonist (RU 486; mifepristone) that inhibits or blocks the function of GC. This treatment increases the production of pro-inflammatory cytokines, including TNF-α responses to LPS stimulation [30], which drive pathobiont-protective Th1 and Th17 responses [14,85]. In the rat model of experimental periodontitis, chronic treatment with RU486 reduced the destruction of periodontal tissue in high-stress responding and periodontitis-susceptible animals [30]. High levels of GCs are common in diabetic patients [124,125], who are susceptible to periodontitis [126], and chronic treatment with RU 486 prevents the worsening of experimental periodontitis in a diabetic rat model [27]. Psychological and physical stress are significant triggering factors for the onset of both type 1 diabetes and type 2 diabetes [127]. In chronically stressed individuals, higher secretion of GC influences glucose metabolism by promoting gluconeogenesis in the liver, suppressing glucose uptake (adipocytes and skeletal muscles), promoting lipolysis in adipocytes, suppressing insulin secretion, and inflicting insulin resistance and inflammation [128].

Taken together, emotional stress and pathobionts, including abnormal growth of Gram-negative bacteria in dental plaque [18,19], activate the CRH-producing parvocellular neurons in the PVN of the hypothalamus, resulting in increased release of GCs into the blood. These brain-controlled mediators dampen strong pro-inflammatory responses by binding to nuclear sensors (MRs and GRs) in the cytoplasm of immune cells. In patients responding to danger signals with an overly strong HPA axis response, typical in diabetics [127,129], melancholic (typical) depression [130], and Alzheimer’s disease [131], the immunological defence against Gram-negative bacteria and other pathobionts is reduced [18].

### 3.7. Hippocampus—Determining Learning Outcomes, Memory, Navigation, Childhood Experiences, the Strength of Stress and Immune Responses, Periodontal Health and Disease

Peripheral stimulation with Gram-negative bacterial LPS activates neurons in the hippocampus [132], located in the brain’s temporal lobe (Figure 1). The hippocampus plays a vital role in both learning and memory, including short- and long-term memory, remembering our body’s position to nearby objects, experiences, and verbal recall. Furthermore, the hippocampus functions as an internal “GPS,” aiding environmental navigation [133]. Alzheimer’s disease is linked to severe periodontitis [134,135] and is characterised by memory loss and cognitive dysfunctions due to neuro-inflammatory damage in the hippocampus [133]. In the rat model of experimental periodontitis, lesions in hippocampal regions related to learning and memory accelerated disease progression [136].

The hippocampus plays a vital role in immune regulation, as the number of GRs in the hippocampus has been found to significantly influence the strength with which stress responses are dampened [11,12,18,137]. The number of hippocampal GRs expressed is genetically determined, and a high number of these sensors is linked to weaker stress responses, while a low number is associated with stronger stress responses [11,12]. However, the gene (*NR3C1*) that codes for this sensor is particularly sensitive to epigenetic modification by the inner and outer environment, particularly just before and after birth [18,138,139,140]. In early life, the internal and external environment can downregulate and upregulate the *NR3C1* gene in the hippocampus through epigenetic mechanisms (DNA methylation and de-methylation), making an individual’s stress responsiveness weaker or stronger. An experimental model in rats demonstrated that early life experiences can make an individual more resistant or susceptible to periodontitis via these mechanisms [18,22,23,24,25,26].

In humans, premature birth and low birth weight are periodontal risk factors [141,142,143]. Premature birth and low birth weight increase the methylation level in the promoter of the *NR3C1* gene, resulting in a reduced number of GRs in the hippocampus [144,145]. Subsequently, this type of stress at birth programmes an individual to stress hyper-responsiveness [146,147,148]. Very early adverse life experiences, leading to stress hyper-responsiveness, also increase the risk for mood disorders in adulthood, including melancholic depression [149], which is a risk factor in periodontitis [150,151,152,153,154,155,156,157]. Furthermore, in a rat model mimicking melancholic depression in humans, the progression of experimental periodontitis was more severe, and they showed a stronger stress response and decreased expression of GRs in the hippocampus. These effects were reversed when the animals were chronically treated with a stress-reducing and anti-depressive drug [28]. Thus, epigenetic modification of the stress system in early life, a phenomenon known as developmental epigenetic programming of health, disease, and behaviour [144,146,147], appears to be an essential and genuine, yet underexplored, risk factor for periodontal disease. As discussed in Section 5, these neurons interact in an “ensemble” with serotonin-producing neurons in the dorsal raphe nucleus (Section 3.8) and dopamine-producing neurons in the ventral tegmental area (Section 3.9), playing a vital role in active coping behaviour [158], which is essential for periodontal health [159].

### 3.8. The Dorsal Raphe Nucleus—Regulating Stress and Immune Responses Through Serotonin and Is Crucial for the Onset of Depression and Periodontitis

The dorsal raphe nucleus (Figure 1) contains serotonin-producing neurons with projections throughout the brain and plays a role in regulating various brain functions (Figure 2). These include mood, sleep, appetite, visceral pain [160,161], immune regulation [162,163,164,165,166,167], microbiota composition [168], and cognitive functions [160,161]. Abnormally low serotonin levels are linked to anxiety and depression [100]. Serotonin, also known as 5-hydroxytryptamine (5-HT), is the most frequently targeted pharmacological agent for treating anxiety and depression [169], both of which are periodontal risk factors [150,151,152,153,154,155,156,157]. Selective serotonin reuptake inhibitors (SSRIs) increase the signalling in dorsal raphe nucleus serotonin-producing neurons, alleviate anxiety and depressive moods and inhibit the progression of experimental periodontitis in rats [18,170,171]. Thus, the dorsal raphe nucleus is regarded as crucial for the onset of depression, and it is now also recognised as necessary for the onset of periodontitis [18]. Injection of LPS or formaldehyde into this nucleus induces depression-like behaviour and concurrent neuroinflammation in mice [172].

Under stress, serotonin produced in the dorsal raphe nucleus helps suppress the stress response by inhibiting the production of the peptide hormone CRH by neurons in the amygdala and the PVN neurons in the hypothalamus (Figure 1) [173]. Low serotonin levels skew the balance between pro-inflammatory and anti-inflammatory adaptive responses (the Th1/Th2 and Th17/Treg balance) towards the Th2 and Treg sides, thereby reducing the immunological defence against Gram-negative bacteria and other pathobionts. In contrast, high serotonin levels have the opposite effect, thereby boosting the immunological defence against pathogen [100,162,163,164,165,166,167,168]. The accumulation of LPS in gingival pockets in rats [19], mimicking an abnormal growth of Gram-negative bacteria observed in active human periodontitis, along with restraint stress in rats [174], accelerates the progression of experimental periodontitis.

In contrast to Gram-negative bacteria and other microorganisms with invasive properties that activate the brain’s stress system, some “kind” bacteria, including the non-pathogenic Gram-positive bacterium *Mycobacterium (M.) vaccae*, along with a vaccine derived from this bacterium, activate serotonergic neurons in the dorsal raphe nucleus [175]. This diminishes the stress response and has antidepressant effects during stressful emotional experiences. This vaccine was found to inhibit the progression of experimental periodontitis in rats [32,33]. Therefore, the inhibitory effect of the *M. vaccae* vaccine on the development of periodontitis may partly result from this bacterium’s stimulatory influence on serotonergic neurons in the dorsal raphe nucleus [18].

In addition to being produced by neurons and serving as a neurotransmitter in the central nervous system, serotonin is synthesised by enterochromaffin cells in the gastrointestinal tract and functions as a hormone [167,168]. These gut-resident cells produce around 90% of the body’s total serotonin, exceeding the amount produced by serotonin-generating neurons in the brain. Enterochromaffin cells, located adjacent to the epithelium of the intestinal villi, release the serotonin they produce into the bloodstream, acting as a hormone throughout the body. The serotonin hormone regulates several physiological processes in peripheral tissues by binding to their corresponding sensors in tissues and immune cells. Almost all immune cells express sensors that respond to serotonin. Low levels of serotonin in the blood are linked to a weakened immune defence against pathogens and mood disorders, whereas high levels have the opposite effect [167,168].

### 3.9. The Ventral Tegmental Area—Involved in Rewards, Positive Emotions, Stress Responses, and Enhancement of Immune Defence Against Gram-Negative Bacteria and Cancer Cells by Dopamine

The ventral tegmental area is in the midbrain (Figure 1) and is a central part of the reward system. It governs diverse behaviours by employing the neurotransmitter dopamine as a signalling molecule [176]. When animals and humans anticipate or receive rewarding stimuli, they develop positive feelings and behaviours, along with pro-inflammatory immune responses. This phenomenon was demonstrated in mice, where stimulation of dopamine-producing neurons in the ventral tegmental area induces positive emotions and enhances immunological defence against Gram-negative bacteria and cancer cells [177,178]. In another experiment, stimulation of the same dopamine-producing neurons reduced the levels of IL-6 in response to peripheral LPS stimulation [179]. This cytokine is enhanced in depressed individuals and animal models of depression of the melancholic type. IL-6 is also enhanced during sickness due to infection [179]. While activation of dopamine-producing neurons in the ventral tegmental area stimulates pro-inflammatory and inhibits anti-inflammatory cytokine secretion by immune cells, cytokines from immune cells can affect dopamine production and release from these neurons. For example, the anti-inflammatory cytokine IL-10, which plays a key role in down-regulating peripheral and central pro-inflammatory responses, activates dopamine neurons in the ventral tegmental area by binding to IL-10 receptors on these cells [180]. Chronic stress-induced activation of the dopamine-producing neurons in the ventral tegmental area, which project to serotonin-producing neurons in the dorsal raphe nucleus, facilitates increased active coping [181]. This finding has helped to illuminate our findings that individuals with well-developed coping strategies to manage daily stress are resistant to periodontitis and that those with poorly developed coping strategies are highly susceptible [159].

Together, these studies demonstrate that activating dopamine-producing neurons in the ventral tegmental area makes an individual more resistant to infections by reducing stress responses to danger signals.

### 3.10. The Insular Cortex—Roles in Decision-Making, Information Storage, Immune Responses and Body Homeostasis

The insular cortex is part of the cerebral cortex, folded deep within the lateral sulcus in each hemisphere of the brain (Figure 1). These brain structures are closely connected to sensory, emotional, motivational, and cognitive systems, playing a significant role in decision-making and regulation of the body’s homeostasis. Additionally, the insular cortex is the primary cortical site of interoception, that is, sensing the body’s physiological state by integrating information regarding bodily sensations, including inflammation, infection, pain, hunger, and other warning signals from external and internal sources of the environment [182,183].

Understanding this brain region has been a challenge due to its location. However, interest in its function has increased significantly with the introduction of fMRI techniques. These techniques have revealed the existence of four distinct functional regions within the insular cortex: a sensorimotor region in the mid-posterior insula, a central-olfactory-gustatory region, a socio-emotional region in the anterior-ventral insula, and a cognitive anterior-dorsal region [184]. A double-blind, placebo-controlled study involving humans using fMRI discovered that LPS activates and significantly impacts inflammation and mood in the anterior and posterior insula [185]. Specific immune responses may be stored and “remembered” in this brain region [186]. These studies suggest that information about immune responses arriving at the cNTS, and from there to the rVLM, activates neurons in the insular cortex (Figure 2), which, like memory T and B cells in the immune system, may “learn” from initial exposure to pathogens and the immune signals released. This means they may react more quickly and effectively to the same pathogen upon subsequent exposure, aiding in infection prevention. Since brain cells are interconnected, the information about previous immune responses from the insular cortex may be more specific than that stored in memory T and B cells [185,186]. Like other brain structures activated by danger signals, the insular cortex is a structure where emotional stress and systemic LPS exposure activate neurons and glial cells, including microglia cells and astrocytes, inducing symptoms of sickness and tissue-destructive inflammation in the activated brain areas [187].

### 3.11. The Medial Prefrontal Cortex—Controlling Stress and Immune Responses and Playing a Role in Shift Work, Sleep Loss, Sleep Deprivation, and Periodontitis

The prefrontal cortex encompasses the frontal portion of the cerebral cortex (Figure 1). It is associated with higher cognitive functions such as decision-making, motivation, problem-solving, planning, and attention. Furthermore, the prefrontal cortex is part of the network that regulates stress and immune responses (Figure 2). As for other brain regions activated by stressful stimuli, including peripheral injection of Gram-negative bacterial LPS and emotional stress, microglia, astrocytes, together with neurons are activated, resulting in neuroinflammation if excessive over time [188,189,190]. The LPS-activated neurons in the medial prefrontal cortex regulate stress responses, the immune system network, and social behaviour by utilising dopamine as a signalling molecule. As for the hippocampus, signals from the prefrontal cortex play a crucial role in immune regulation, as they dampen the strength of stress responses. The frontal cortex reduces the stress response by cognitive self-regulation (Figure 2) [11,12]. The strength of the stress system is crucial for immune regulation and plays a vital role in maintaining periodontal health and preventing disease [18].

Research has shown that the prefrontal cortex also plays a vital role in sleep loss and sleep deprivation among shift workers [190], another risk factor for periodontal health [191,192,193]. This relationship was also seen in a rat model simulating sleep deprivation, where the progression of experimental periodontitis was more severe [194]. In contrast, melatonin treatment, the hormone that regulates the body’s sleep–wake cycles, inhibited the increased periodontal tissue destruction induced by restraint stress [195]. Loss of sleep and sleep deprivation, or depression, are stressors that activate the stress system and its regulatory network [190]. They also increase anxiety and depressive symptoms in healthy individuals [196,197,198,199,200]. Chronic sleep deprivation aggravates LPS-induced anxiety, depression, and cognitive impairment, which was associated with changes in pro-inflammatory cytokines and proteins involved in stress regulation [200].

Sleep loss and sleep deprivation resulted in abnormal growth of pathobionts in the gut microbiota [201,202]. In periodontology, it is well accepted that an abnormal growth of pathobionts initiates periodontitis. Peripheral LPS stimulation, mimicking dysbiosis, increased the inflammation in the medial prefrontal cortex in an animal sleep deprivation model, which was more pronounced in older individuals than in younger ones [203]. In another animal sleep deprivation model, it was found that microglia were activated in the prefrontal cortex, and that the pro-inflammatory cytokine TNF-α released by these cells acted as a signalling molecule that triggered anxiety behaviour induced by sleep deprivation [204]. When mice were intraperitoneally injected with LPS, mimicking systemic Gram-negative infection, they showed neuroinflammation in the prefrontal cortex and cognitive dysfunction, with impairments in reference memory [205].

Alongside advanced AIDS (acquired immunodeficiency syndrome) and exposure to extreme stress, such as serving as soldiers in a war zone, sleep loss and deprivation are also risk factors for acute necrotic ulcerative gingivitis (ANUG) and acute ulcerative periodontitis (ANUP) [206], diseases that are very rare and characterised by pathobionts infecting the gingiva and periodontium. These rare gingival and periodontal conditions have been observed in military populations throughout history [207]. The severity of life stressors and an individual’s stress responsiveness correlate with the severity of periodontitis [18]. This suggests that sleep loss and deprivation are extreme stressors and key risk factors for periodontitis.

### 3.12. The Cerebellum—Regulating Body Balance, Stress and Immune Responses, Emotions, Learning, and Memory

The cerebellum (Figure 1 and Figure 2) is primarily recognised for its crucial role in motor coordination and balance. It also consistently engages in sensory activities, speech and language, motor and spatial memory, and psychological functions [208,209]. The cerebellum may also control various regions of the hypothalamus involved in stress regulation [209]. Since activities in the cerebellum influence stress responses, they may also play a role in the nervous and hormonal control of the immune system functions [210]. Various studies have reported that the cerebellum regulates the hypothalamus through a feedback neural circuit, which bi-directionally connects areas of the hypothalamus, with corresponding areas of the cerebellum (Figure 2) [210]. LPS activates glial cells in the cerebellum, leading to inflammation, which can be prevented by drugs that diminish the stress response [211].

Like the hippocampus, amygdala, and insular and frontal cortices, the cerebellum stores acquired information, a primary function necessary for learning and memory [211]. Emotional responses, such as fear, stress, and depression, adversely affect these cognitive functions. In contrast, optimistic and happy emotions have a positive influence on long-term memory. These studies have also revealed that the brain’s right hemisphere regulates negative emotions while the left hemisphere regulates positive emotions [212]. Like emotional stress responses, immune activation by pathogenic microorganisms, most exemplified by Gram-negative LPS or IL-1β, leads to deficits in learning and memory. These studies demonstrate that LPS or IL-1β exposure increased anxiety and depressive mood and that drugs with anxiolytic and antidepressant effects inhibit or reverse these effects, as well as deficits in learning and memory [213,214,215]. Immunoception has recently been introduced to describe the brain’s bidirectional monitoring and regulation of immunity [216]. Thus, it is quite possible that the cerebellum is involved in regulating and coordinating the Th1/Th2 balance and the Th17/Treg balance. It is, however, unlikely that the ability to learn and remember is seriously affected in patients with common chronic periodontitis, since the gingiva and the periodontium are not infected in this condition, and *P. gingivalis* LPS is a weak signal compared to LPS from the gut bacteria *E. coli*, as explained in Section 3.1.

## 4. Neuroinflammation, Depression, Neurodegenerative Diseases, Oxidative Stress, and Periodontitis

Adverse early life events and psychosocial stressors in adulthood activate non-neural cells known as glial cells in specific brain structures alongside neurons [217]. They are also activated during trauma and infection, and when local tissues are exposed to pathogenic microorganisms, including Gram-negative bacteria and their products, and cytokines from robust immune responses [218]. While neurons transmit information through nerve impulses and release neurotransmitters, glial cells do not generate electrical impulses. Instead, they produce and release pro-inflammatory cytokines such as TNF-α, IL-1β, IL-6, and IL-33, along with anti-inflammatory cytokines like IL-10 and transforming growth factor (TGF)-1β, communicating with other brain cells. Their main role is to support, correct, and protect the neurons [219,220]. Stress, infection, or other signals of danger trigger inflammation in the activated areas of the brain. This is a crucial element of normal neurotransmission within the CNS. These inflammatory responses in the CNS are referred to as neuroinflammation [219,220,221,222,223].

Like inflammation in the gingiva, inflammation in activated brain regions, caused by danger signals of any kind, real or imagined, may be clinically silent or manifest as either non-destructive or destructive to the surrounding cells, which are neurons in the brain. To differentiate between the two, “para-inflammation” has been introduced to describe a non-pathological, homeostatic response without neuronal damage, whereas “neuroinflammation” denotes a pathological state that harms neurons [221]. Thus, neuroinflammation results from persistent psychological stress and/or infection caused by pathogens that have infected tissues, provoking an inflammatory response in the brain and can lead to neurodegenerative diseases such as Alzheimer’s and Parkinson’s. In Alzheimer’s, glutamate-producing neurons in the hippocampus are mainly affected, while in Parkinson’s, noradrenaline-producing neurons in the locus coeruleus and dopamine-producing neurons in the substantia nigra are damaged [224,225,226,227,228,229]. These neurons are especially susceptible to neurodegeneration induced by Gram-negative bacterial LPS. Neuroinflammation also plays a vital role in brain damage during sepsis and can lead to various neuropsychiatric symptoms in both humans and animals, including mood changes similar to clinical depression, called sickness behaviour [2,211]. The administration of LPS is now widely used as a model for melancholic (typical) depression and neurodegenerative diseases [229]. LPS-induced neuroinflammation is associated with cognitive impairments connected to the prefrontal cortex, leading to symptoms of sickness behaviour [2,230,231].

Injecting *P. gingivalis* into the gingiva [232] or intraperitoneally [233] leads to neuroinflammation. The antidepressant imipramine inhibits neuroinflammation in the hippocampus, induced by LPS from *P. gingivalis,* injected intraperitoneally in mice [234]. This drug also inhibited the progression of periodontitis in mice induced by LPS from the periodontal pathobiont *A. actinomycetemcomitans*, injected into the gingival tissues, as well as reduced the enhanced tissue destruction caused by a high-fat diet, a stressor replicating metabolic syndrome (MetS) [171]. Daily administration of another periodontal pathobiont, *P. gingivalis*, on the gingival surface downregulates brain-derived neurotrophic factor (BDNF) in hippocampal astrocytes, contributing to sickness behaviour [235]. BDNF is a growth factor that plays multiple roles in the nervous system and is crucial for neurogenesis (the development of new neurons) in the adult hippocampus. Emotional stress and infection can impair this process, affecting learning and memory [235]. BDNF also strengthens neuronal synaptic plasticity, i.e., the ability of synapses to change their strength in response to activity, enhancing learning and memory [236], and has therapeutic potential in depression and neurodegeneration [237]. A recent experiment showed that forced treadmill exercise reduced symptoms of depression and enhanced cognitive function in rats that were intraperitoneally injected with LPS, through its influence on brain-derived BDNF expression levels in the hippocampus [238].

In the rat periodontitis model, damaging specific hippocampal regions involved in learning and memory accelerated the disease progression [136]. A low number of GRs in these neurons results in stress hyper-responsiveness and an increased susceptibility to periodontitis. This indicates that reduced levels of GRs in hippocampal neurons may be responsible for elevating the risk of periodontitis. Gingiva is not infected in common chronic periodontitis, only in the rare forms (ANUG and ANUP) [206]. Consequently, injecting LPS or live Gram-negative bacteria into the gingiva or intraperitoneally does not imitate common chronic periodontitis, but rather the rare aggressive forms. The silk ligature-induced periodontitis model, which promotes the growth of pathobionts between the silk fibres in gingival pockets, is therefore more suitable for mimicking common chronic periodontitis [19].

Neuronal death in neurodegenerative disorders is primarily attributed to apoptosis caused by oxidative stress, mitochondrial dysfunction, and disrupted calcium homeostasis [239,240,241]. Like macrophages and polymorphonuclear leukocytes (PMNs/neutrophils) in the gingiva, microglia in the brain kill microbes, remove microbial components, including Gram-negative LPS, and can damage tissue cells by phagocytosis [242]. During phagocytosis by microglia and other phagocytic cells, matrix metalloproteinases and reactive oxygen species are produced in a process called oxidative stress. Oxidative stress caused by neutrophils in gingiva and periodontal tissues is responsible for soft tissue destruction in periodontitis [18]. It is now accepted that excessive activation of microglia in the central nervous system, induced by danger signals, including LPS from *P. gingivalis* injected into the gingiva [243], causes mitochondrial dysfunction mediated by neuroinflammation through oxidative stress [244]. Thus, oxidative stress is responsible for the pathogenesis and progression of both periodontitis and neurodegenerative diseases.

Oxidative stress generates formaldehyde (CH_2_O) [171]. Formaldehyde is predominantly recognised for its role in the preservation of dead bodies and body parts when utilised as a solution. Recent research has identified endogenous formaldehyde as significantly contributing to neuroinflammation and the onset of depression in animals subjected to both acute and chronic LPS injections. Supplementation of the coenzyme Q10, an endogenous formaldehyde scavenger, diminished formaldehyde accumulation and neuroinflammation in the midbrain of mice induced by systemic LPS injection [171], and inhibited the progression of periodontitis in animal experiments [245]. The effect of coenzyme Q10 on periodontitis in humans is not apparent [246]. However, the progression of periodontitis occur when an individual is exposed to danger signals of any kind and illnesses that induce persistent stress hyper-responsiveness during specific periods, such as episodes of severe anxiety, melancholic depression, severe chronic pain, general infections, obesity, hypertension and cardiovascular diseases, certain types of cancer, neurodegenerative diseases like Alzheimer’s, or in diabetic patients when blood sugar levels are elevated [18]. When an individual encounters such danger signals, the unusually high secretion of catecholamines and GCs fails to adapt to the danger signal that activates the stress response [11,12,149]. Thus, dietary supplements that reduce oxidative stress, such as coenzyme Q10, may be effective solely when the periodontal breakdown is active.

Oxidative stress and neuroinflammation are key factors in the depression of the melancholic type [247]. In the depressive phase of patients with bipolar disorder, an abnormal growth of pathobionts has been shown [248]. Dysbiosis is also a significant characteristic of the gut in patients with this type of depression [249,250]. Melancholic (typical) depression is a periodontal risk factor [18,150,151,152,153,154,251]. This type of depression may be triggered by psychological stressors such as the loss of a loved one, divorce, bankruptcy, interpersonal stress, and social rejection [155], which are factors associated with stress hyper-responsiveness and increased susceptibility to periodontitis [18]. In this disorder, depression and neuroinflammation interact with a hyperactive HPA axis, low serotonin levels, and a reduced number of neurons and GRs in the dentate gyrus of the hippocampus [18,157,252,253,254,255]. As noted earlier, pro-inflammatory adaptive (Th1 and Th17) immune responses, which protect against pathobionts and, consequently, against periodontitis, are weaker in patients with depression of the melancholic type. Importantly, individuals with atypical depression often exhibit low stress responses [256,257,258,259]. This is commonly observed in patients with conditions such as post-traumatic stress disorder (PTSD) and chronic fatigue syndrome, and is frequently associated with a history of trauma, including childhood sexual abuse [257]. Due to differences in stress responsiveness, typical and atypical depression display opposite patterns in TNF-α, Th1, and Th17 responses [258,259] and consequently in susceptibility to periodontitis [18]. It is therefore important to differentiate between these two types of depression, not to rely on self-reported depression symptoms.

## 5. Psychological Challenges, Coping Strategies, Periodontal Health and Disease

A comprehensive cross-sectional epidemiological study conducted in Sweden, involving individuals aged 50 to 80, revealed that a person’s ability to cope with stressful stimuli (coping behaviour) significantly influences periodontal health and disease. Those with poorly developed coping strategies for managing daily stress exhibited more severe periodontitis than average, whereas those with well-developed coping strategies had less severe periodontitis. The same study also showed that smokers have more severe periodontitis compared to non-smokers, and being a widow or widower is a significant risk factor for periodontitis [158]. An association between poor coping strategies to manage stressful situations and severe periodontitis has also been verified in other studies [260,261,262]. This is particularly evident in different types of adverse life events, especially those leading to severe anxiety [263], and depression of the melancholic type [18,150,151,152,153,154,251].

Given today’s shared understanding of the aetiology of periodontitis, the relationship between severe periodontitis and adverse life events remains challenging to grasp [264,265], and how an individual’s coping strategy in response to these stressors may influence their susceptibility or resistance to periodontitis. However, coping is a conscious effort to solve problems by minimising stressful conflicts [266,267,268]. It is a term used specifically for the conscious and voluntary mobilisation of actions. It differs from “defence mechanisms”, which are subconscious or unconscious adaptive responses to reduce or tolerate stress. In other words, active or well-developed coping strategies are behavioural responses that minimise physical, psychological, or social harm and relate to stress resilience [269], i.e., the term used to describe the ability to overcome stress. Thus, coping is a concept that describes cognitive and behavioural strategies for managing situations or emotions that are either internally or externally overwhelming or stressful. Individuals with poorly developed coping strategies may respond to specific danger signals (stressors) with too-strong stress responses, whereas those with improved coping skills respond with appropriate stress responses. Based on animal experiments, individuals who respond to stressors with extreme stress reactions show more severe periodontitis, regardless of whether the stress response is genetically determined, altered by very early life experiences through epigenetic mechanisms, or triggered in adulthood by factors and diseases associated with severe periodontitis in humans [18]. Thus, the severity of periodontitis highly depends on the individual’s ability to cope with the stressful situation.

The mechanisms in the brain that contribute to the general coping style are divided into several domains, including those associated with impulse control, emotional reactivity, and the processing of danger and reward. Furthermore, the mechanisms that regulate the release of serotonin are essential in orchestrating overall coping style [268,269,270]. Consequently, the neurotransmitters serotonin and dopamine play a significant role in stress management, influencing interconnected bodily functions that impact both health and disease.

## 6. Microbiota–Immune–Brain Interactions and Clinical Consequences

In this review, the localisation, organisation, processing, and regulation of immune information by the brain, along with its overarching control over immune responses, have been assessed.

One of the primary functions of this two-way communication between the microbiota/immune system and the brain is to dampen and regulate strong pro-inflammatory responses, such as TNF-α responses, triggered by pathobionts. Persistent hyper-responsiveness of the stress system and the resulting excessive dampening of TNF-α responses are identified as a biological link between periodontitis and its associated risk factors and diseases [18]. In individuals with periodontitis, the pro-inflammatory and anti-inflammatory adaptive balances (the Th1/Th2 and Th17/Treg balances) are skewed towards the anti-inflammatory (Th2 and Treg) side by these mechanisms [18]. This protects against Gram-positive bacteria and other non-invasive symbionts but not against Gram-negative bacteria and other pathobionts. Consequently, there is a predominance of *P. gingivalis* and other pathobionts in the microbial biofilm at the gingival margin, increasing the risk of developing periodontitis. In contrast, individuals who display inadequate stress responses to a danger signal exhibit a weaker dampening of TNF-α responses, resulting in excessively strong pathogen-protective Th1 and Th17 responses, which lead to increased gingival inflammation (gingivitis) and improved resistance to periodontitis [18]. Thus, in patients with gingivitis, the number of pathobionts in dental plaque, which initiate periodontitis, is lower than in healthy individuals. Therefore, the long-held belief that gingivitis is the precursor to periodontitis and that managing gingivitis is the primary method of preventing periodontitis [271,272,273] may not be valid. Consequently, we must reconsider how to prevent and treat periodontitis. Reducing dental plaque in patients with gingivitis can decrease inflammation; however, it does not prevent the development of periodontitis. On the other hand, reducing dental plaque in patients with active periodontitis lowers the number of pathobionts and disease progression. Since the disease does not develop continuously but instead occurs when stress responses are extreme and pathobiont-protective immune responses are weak, it is important to identify these periods to prevent and minimise the development of periodontitis.

Minimising risk factors, such as nicotine exposure, disrupted blood sugar control, stress during pregnancy, shortly after birth, and in adulthood, as well as impaired ability to cope with stressful experiences, causing anxiety and depressive feelings, make these approaches promising for preventing severe periodontitis. Since the childhood environment can permanently influence an individual’s genetic response to stress through epigenetics, affecting susceptibility to periodontitis later in life, as shown in animal studies [18,22,23,24,25,26,138,139,274,275,276], this knowledge is essential for facilitating a better understanding of the differences between individuals and distinguishing vulnerability from resilience to periodontitis. Additionally, other treatments that shift the pro- and anti-inflammatory adaptive immune response equilibrium in the direction of pathogen-protective pro-inflammatory adaptive responses, such as the *M. vaccae* vaccine [18,32,33] and dietary supplementation of beta-1,3/1,6-glucan [18,34], may be the future goal of treatment strategies in patients with active periodontitis.

Reduced pro-inflammatory cytokine responses, such as TNF-α, have been considered beneficial in periodontitis [36,37,38,272]. However, animal experiments show that these responses are beneficial and protective in periodontitis. These studies show that TNF-α responses escalate when a stress low-responding individual is exposed to pathogens. In contrast, TNF-α levels are insufficient to control the growth of pathobionts in the gingival sulcus in those with strong stress responses [18]. Moreover, TNF-α responses are weaker and diminished when dental plaque is reduced at the gingival margin or when the adaptive immune defence against pathobionts is enhanced and strengthened by treatment with immune stimuli, such as the *M. vaccae* vaccine, or dietary supplementation with beta-1,3/1,6-glucan, which promotes pathogen-protective Th1 and Th17 responses [18]. This phenomenon was also found to be evident in rats exposed to nicotine, but via effects on specific acetylcholine receptors [18,29].

Periodontal pathobionts in common chronic periodontitis have been suggested to initiate neuroinflammatory diseases, as Alzheimer’s [227]. However, as emphasised above, the gingiva and the periodontium are not infected during common chronic periodontitis. According to the World Health Organisation (WHO), infection involves pathogenic microorganisms invading and multiplying in body tissues [277]. However, this does not apply to common chronic periodontitis, where pathobionts reside in the dental biofilm rather than in the gingival tissues, and do not cause symptoms of illness, as found during ANUG and ANUP. Furthermore, *P. gingivalis* LPS is a significantly weaker danger signal than that from the Gram-negative gut bacterium *E. coli* [81]. As *P. gingivalis* increases in number in the oral cavity during the development of periodontitis, *E. coli* proliferates in the gut of patients exposed to various danger signals, including psychological stress [278], and their prevalence in the ileal mucosa is increased in chronic inflammatory bowel diseases [279]. The population of these gut bacteria is therefore considerably more harmful to the organism than that found in the oral cavity. They trigger a stress response far more potent than that caused by *P. gingivalis* LPS [83].

While *P. gingivalis* often dominates in common chronic periodontitis, spirochetes, such as *Treponema denticola*, predominates in dental plaque associated with ANUG and ANUP [206]. Their abnormal growth also escalates during substantial stress, such as experienced by soldiers in a war zone [280], sleep loss, sleep deprivation [206]. and advanced AIDS [281,282]. People under prolonged periods of severe anxiety, particularly those with substantial stress (SNS, SAM and HPA axis) responses, are susceptible to infections and cancer, and may experience impaired wound healing [283,284]. In AIDS patients, the human immunodeficiency virus (HIV) attacks the Th cells, especially the Th17 cells [281,282], which are the Th cells that orchestrate the immune response that prevents pathobionts from infecting mucosal tissues. Thus, the extreme tissue load of spirochetes occurs when the stress system is hyperactivated and the adaptive immune system is highly weakened and unable to protect against infection. Therefore, we recommend administering antibiotics to patients with ANUG and ANUP in the initial treatment phase, as well as to those with rapidly progressive periodontitis who exhibit signs of illness. However, we do not recommend using antibiotics to treat common chronic periodontitis. This aligns with the recommendations from other investigators, the WHO, and the European Union (EU), which advise against using antibiotics to treat chronic infectious diseases, particularly those caused by Gram-negative bacteria, to prevent the development of bacterial resistance [285]. This is because antimicrobial resistance poses a significant global health risk and is among the most serious threats humanity faces today. Since the symbiotic dental microorganisms (pathobionts) that initiate periodontitis can be removed by toothbrushing, reducing dental plaque at the gingival margin should be the primary treatment for common chronic periodontitis [286].

General microbiota are essential for developing an adaptive immune system and the overall regulatory mechanisms in the brain that control immune responses, various physiological processes, and emotional behaviour [287,288,289,290,291,292]. Indeed, there is evidence today that the microbiota shapes the adaptive immune system from birth and throughout life [18,293], and that the adaptive immune system, with the help of the innate immune system, shapes the composition and number of microorganisms in the microbiota [18,294]. Furthermore, the microbiota influences the functioning of the brain-controlled homeostatic systems [288] and regulates immune system responses. Pathobionts, such as *P. gingivalis*, may thus be crucial for developing a well-functioning adaptive immune and stress system. Understanding how genetics, epigenetics, and environmental factors influence brain-controlled immunoregulatory mechanisms and immune response encoding will have a significant impact on periodontal health research. Since the microbiota is regarded as a vital organ of the body [295], addressing diseases like periodontitis, caused by microbiota dysbiosis, may be a challenge we face indefinitely. Collaboration between basic and clinical scientists to uncover the interactions of the dental microflora-brain axis is essential for periodontal research.

## Data Availability

Not applicable.
